# The Challenge of Neuropsychiatric Systemic Lupus Erythematosus: From Symptoms to Therapeutic Strategies

**DOI:** 10.3390/diagnostics14111186

**Published:** 2024-06-05

**Authors:** Veena Patel

**Affiliations:** Department of Medicine, Division of Rheumatology, Dell Medical School, The University of Texas at Austin, Austin, TX 78712, USA; veena.patel@austin.utexas.edu

**Keywords:** SLE, NPSLE, antiphospholipid antibodies

## Abstract

Systemic lupus erythematosus (SLE) is a chronic autoimmune condition that can seriously impair multiple organs including the nervous system, causing neuropsychiatric SLE (NPSLE), which encompasses a broad range of symptoms. Pathogenesis is not completely understood but is thought to involve inflammatory and vascular pathways. This comprehensive review discusses the complex nature and heterogeneity of NPSLE and the challenges in diagnosis and treatment that result from it. Diagnosis often requires a multidisciplinary approach with multiple assessments, including laboratory testing, imaging, and neuropsychological evaluations. Current treatments focus on managing symptoms through immunosuppressive and anti-thrombotic therapies tailored to the inflammatory or vascular nature of the specific NPSLE manifestations. This paper emphasizes the necessity for interdisciplinary approaches and further research to enhance diagnostic accuracy and treatment effectiveness. It also highlights the importance of understanding the underlying mechanisms of NPSLE to develop more targeted therapies, citing the need for high-quality studies and novel treatment agents.

## 1. Introduction

Systemic lupus erythematosus (SLE) is a chronic, multiorgan autoimmune disease which can cause internal organ manifestations that can become life-threatening, leading to organ failure or death. SLE can affect many systems of the body, including the central and peripheral nervous system, and can cause psychiatric manifestations. Neurologic manifestations of lupus can lead to poorer quality of life and increased mortality compared with SLE patients without neuropsychiatric (NP) manifestations [1].

Previously, when SLE affected the nervous system, it was termed “lupus cerebritis”, “neurolupus”, or “CNS lupus”, none of which accurately encompass the many types of neuropsychiatric (NP) syndromes SLE can cause. Now, when SLE causes NP symptoms, it is called neuropsychiatric lupus (NPSLE). NPSLE manifestations can be focal or diffuse and can affect the central or peripheral nervous system [2].

Given the heterogeneity of neurologic symptoms and the pathogenesis that can be attributed to SLE not being fully understood, NPSLE can be rather challenging to diagnose. To add to this challenge, these manifestations can occur at the time of diagnosis or at any time during SLE’s disease course, where individual or multiple neurologic syndromes may present in the same individual. In this review, we plan to review important aspects of the disease and talk about future strategies to help diagnose, treat, and understand NPSLE. 

## 2. Clinical Manifestations 

NPSLE encompasses a vast array of neurologic and psychiatric symptoms. The majority of NPSLE symptoms occur at time of diagnosis of SLE or after [3], typically within the first year after diagnosis [4]. The overall prevalence was 56.3%, predominantly affecting CNS (93.1%) rather than PNS (6.9%) [5,6]. NPSLE manifestations such as seizures and psychosis are more common in early-onset SLE (diagnosed < 50 yo), while peripheral neuropathy are more common in late-onset SLE [7].

In 1999, the American College of Rheumatology developed standard nomenclature and case definitions for NPSLE syndromes. Nineteen syndromes were identified and defined, including 12 central nervous system (CNS) and 7 peripheral nervous system (PNS) syndromes [2]. Table 1 lists these syndromes. 

The most common NP syndromes (occurrence rate: >10–20%) were headaches, mood disorders, and mild cognitive dysfunction [3,5,6]. Frequent NP syndromes (occurrence rate: 5–10%) included seizures, cerebrovascular disease, and anxiety. Uncommon syndromes (occurrence rate: 3–5%) include psychosis, peripheral neuropathy, an acute confusion state, and severe cognitive dysfunction, and rare syndromes (<1–2%) include chorea or movement disorders, aseptic meningitis, cranial neuropathy, mononeuritis multiplex, demyelinating syndrome, Guillain-Barre syndrome (GBS), autonomic disorders, myasthenia gravis, and plexopathy [5].

### 2.1. Focal CNS Syndromes

Focal CNS syndromes included in the ACR nomenclature report include aseptic meningitis, cerebrovascular disease, demyelinating syndrome, headaches (including migraines and benign intracranial hypertension), movement disorders (chorea), myelopathy, and seizure disorders. 

Headaches are quite common in lupus patients, with no clear association with disease activity. Migraines and tension headaches are the most reported syndrome, but their prevalence is no different than that of the general population [6]. The entity of a “lupus headache” is a diagnosis of exclusion, but there has not been a mechanism found to specifically attribute this to SLE [8].

Acute ischemic stroke is one of the most common neurologic manifestations in SLE and can affect 3–20% of SLE patients, usually within the first five years of diagnosis. There is a higher risk of all stroke subtypes in patients with SLE. Strokes can be due to a hypercoagulable state caused by antiphospholipid syndrome, emboli from endocarditis, accelerated atherosclerosis, or cerebral vasculitis [9]. Determining the underlying cause is critical to ensure proper management is pursued.

Antiphospholipid syndrome (APS) is an autoimmune disease characterized by specific antiphospholipid antibodies (APLAs)—anti-cardiolipin IgM and IgG, anti-beta2glycoprotein IgM and IgG, and lupus anticoagulant—which cause a hypercoagulable state, leading to venous and arterial thromboses. APS can be a primary disease, but 20–30% of SLE patients are positive for APLAs, and if they are at moderate-to-high titers, then patients are at increased risk of thrombosis even without any history of thrombotic events. APLAs are implicated in the vascular pathway of NPSLE pathogenesis and can cause strokes, venous thromboembolisms, cerebral venous sinus thrombosis, cognitive impairment, peripheral neuropathy, and chorea. APS can cause other manifestations, including obstetric complications and livedoid vasculopathy [9,10,11].

Single tonic-clonic seizures are common and related to SLE disease activity, usually appearing early in the disease’s course, and recurrence is uncommon in SLE patients [4]. If there is recurrence, then it usually happens within the first year [8].

Demyelinating disorders are a rare manifestation of NPSLE, with a prevalence of 0.3–2.7% [9]. Diagnosis can be a challenge as they can closely resemble multiple sclerosis, and there is not a single test that can differentiate between the two diseases. Optic neuritis can also present in SLE patients and can be related to demyelination or axonal loss, but it is most often caused by ischemia. It can occur as in isolation and is usually bilateral in NPSLE [4]. This presentation can be difficult to differentiate from neuromyelitis optica spectrum disorder, especially if occurring in isolation. Fifty percent of SLE patients met the diagnostic criteria for neuromyelitis NMOSD [12]. If there are systemic symptoms present such as rashes, cytopenias, or glomerulonephritis, then the diagnosis may point toward SLE.

Transverse myelitis (TM) is a rare manifestation that is associated with APS as it can be due to thrombotic or ischemic events, but it can also be inflammatory in nature and associated with NMOSD, and thus checking NMO-IgG or AQP4-Ab should be considered in patients presenting with SLE and TM [13].

Other focal CNS syndromes reported in NPSLE include cerebral venous sinus thrombosis, which is most often associated with APS and active SLE. Posterior reversible encephalopathy syndrome (PRES) has also been reported in patients with SLE. Progressive multifocal leukoencephalopathy occurs most frequently with SLE compared with other autoimmune conditions but has also been associated with rituximab use [8,9].

### 2.2. Focal Peripheral Nervous System Syndromes

Focal PNS syndromes include peripheral neuropathy, acute inflammatory demyelinating radiculoneuropathy, autonomic disorders, mononeuropathy (single or multiplex), myasthenia gravis, cranial neuropathies, plexopathy, and polyneuropathy [2].

A study of 1224 lupus patients showed that the most common PNS manifestations were polyneuropathy, cranial neuropathy, and single or multiple mononeuritis. The average age of onset of SLE was higher in patients with PNS involvement than the controls, and they were more likely to have higher disease activity, hypertension, and livedo reticularis occurrence [14].

Acute inflammatory demyelinating radiculoneuropathy most commonly presents as Guillain-Barre syndrome. The most common cranial neuropathies are III, V, VI, and VII. Peripheral neuropathy is quite common and be challenging to attribute to SLE. SLE patients also have high rates of small fiber neuropathies [15].

### 2.3. Diffuse CNS Syndromes

Diffuse CNS syndromes include an acute confusional state, anxiety disorders, cognitive dysfunction, mood disorders, and psychosis. These were most difficult for the ACR committee to determine whether they were NP manifestations or psychological reactions to stress or coping with chronic systemic illness.

Mild-to-moderate cognitive impairment has an estimated prevalence of 14–95%, while severe cognitive impairment has a much lower prevalence of 3–5%, and progressive cognitive decline is rare [8,16]. The most commonly affected domains include attention, visual and verbal memory, executive function, and psychomotor speed. Factors related to severe cognitive dysfunction include hypertension, APLAs, and presences of strokes and lesions in MRI that correlate with those affected in neuropsychologic testing [8].

Psychosis occurred either in the year prior to or within 3 years of SLE diagnosis. Factors associated with lupus psychosis are previous SLE NP events, being of the male sex, a younger age at SLE diagnosis (per 10 years), and African ancestry [17]. Cognitive impairment, psychosis, and depression are associated with many antibodies, including anticardiolipin, but none have been shown to be diagnostic [18].

The ACR nomenclature faced criticism for low specificity of diagnosing NPSLE with the 19 syndromes identified in their report. Manifestations such as headaches, mood disorders, anxiety, and mild cognitive dysfunction are common but do not usually reflect overt central nervous system (CNS) lupus activity. In a study conducted by Ainaila et al. [19], these manifestations were excluded from revised criteria along with polyneuropathy without electrophysiological confirmation, which resulted in a reported NPSLE frequency decrease of half, and the specificity of the ACR nomenclature increased from 46% to 93% [19,20].

## 3. Diagnosis

NPSLE is challenging to diagnose because none of the syndromes have findings specific to SLE. With the lack of specific diagnostic biomarkers or imaging findings, diagnosis is still mainly based on expert opinion. Along with expert judgment, clinical symptoms, autoantibody positivity, imaging findings, and exclusion of other causes are taken into account to diagnose NPSLE. Often, a multidisciplinary approach including neurology, psychiatry, vascular specialists, hematology, and neuroradiology is necessary to aid in diagnosis.

Risk factors for NPSLE events include generalized SLE disease activity, previous severe NPSLE manifestations (especially for seizure disorders and cognitive dysfunction), and positive APLAs (especially for cerebrovascular disease, seizures, and chorea) [4,5]. The male sex, a history of NPSLE events, and NP damage have also been reported as risk factors for impending flare-ups of NPSLE [21].

### 3.1. Diagnosing SLE

NPSLE may occur after SLE has been diagnosed or at the onset of SLE diagnosis. Sometimes, NPSLE manifestations may be the only active symptom at the time of presentation, which can add to the diagnostic challenge.

To diagnosis an individual with SLE, one must have certain clinical manifestations along with specific autoantibody positivity. SLE presents a diagnostic challenge, as symptoms are often vague, may have a slow, insidious onset, and may not all appear at the same time. Our screening laboratory test for SLE, the antinuclear antibody (ANA), is also rather nonspecific and can be positive in many other conditions and healthy individuals. Classification criteria for SLE, such as the SLICC [22] or EULAR [23], are helpful guides to use for assessing symptoms and lab findings, but it is important to remember that they are not diagnostic criteria. While the ANA is non-specific, it is required for SLE diagnosis, and more specific antibodies, including the anti-Smith antibody, double-stranded DNA (dsDNA), and hypocomplementemia (C3/C4), can be present in SLE.

#### Assessing for SLE Flares

It is important to assess for generalized disease activity in SLE, as active SLE is a known risk factor for many NP manifestations [24], and NPSLE is often seen in 40–50% of patients with generalized SLE activity [4]. SLE flares are characterized by active clinical symptoms, which can be the same symptoms the specific patient generally presents with or can be a new symptom as well. Laboratory tests which may help to assess disease activity or if a flare is happening assess positive dsDNA or low C3/C4 values. It is also important to assess cytopenias and kidney function with urinalysis to measure proteinuria and red cell casts. Discussing with the patient what their typical flare symptoms are and checking the laboratory tests mentioned will provide insight into whether a patient is having a flare of SLE.

### 3.2. Diagnosing Antiphospholipid Syndrome

The presence of moderate-to-high titer APLAs is a risk factor for many NPSLE manifestations, and they have been implicated in the vascular pathogenesis pathway. While confirmed diagnosis of APS is not necessary to confer a risk of NPSLE, some patients may end up concurrently being diagnosed with APS based on labs and clinical findings.

The 2023 ACR/EULAR APS classification criteria provide a good guide as to what autoantibodies and symptoms are seen in APS [10]. APS causes venous or arterial thromboses in the setting of positive antiphospholipid antibodies at moderate-to-high titers, being positive on multiple occasions. Included in the criteria are lupus anticoagulant, anti-cardiolipin IgG or IgM, and beta 2 glycoprotein IgM or G [11]. When considering NPSLE, especially ischemic manifestations, checking APLAs may help.

### 3.3. Diagnosing NPSLE

Since there is no single diagnostic test to diagnose NPSLE, the first step is to exclude secondary causes of neurologic conditions such as infections, metabolic or endocrine disorders, adverse drug reactions, and malignancy. The work-up should be tailored to the specific disease manifestation that the patient is presenting.

#### 3.3.1. Laboratory Testing

With serum lab testing, we recommend checking whether to rule out thyroid disease and vitamin diseases and testing for infections by including blood cultures, if appropriate, a complete blood count with a differential, comprehensive metabolic panel, urinalysis with microscopy, and a random urine protein/creatinine ratio. These basic lab tests will also give information about generalized lupus activity (cytopenias, kidney dysfunction with proteinuria, or red cell casts).

If there is already a diagnosis of SLE, then checking the antibodies that fluctuate with disease activity—dsDNA, C3, and C4—is also helpful. If there is a patient suspected to have a new diagnosis of SLE, then checking for ANAs and anti-Smith antibodies and considering checking other extractable nuclear antigens based on clinical symptoms, including anti-U1RNP, anti-Ro, and anti-La antibodies, would be helpful.

All patients who are suspected to have NPSLE should have their APLAs checked, since +APLAs are a risk factor for certain NPSLE manifestations. There is limited diagnostic utility with anti-ribosomal P antibodies, and thus we would avoid checking for these during workup.

Lumbar puncture and cerebrospinal fluid (CSF) testing are important for ruling out CNS infection. Checking cell counts and cultures and PCR testing for herpes simplex virus, JC virus, and other infections is important. NPSLE can cause mild, nonspecific abnormalities in the CSF, including elevated WBC and protein levels with low glucose. The IgG index also increases by up to 75% in diffuse NPSLE, but again, this is not specific [4,5].

#### 3.3.2. Imaging

Conventional MRI for the brain and spine (depending on symptoms) is the test of choice in NPSLE and can be useful for excluding other pathologies, although 50% of NPSLE cases do not have detectable abnormalities [5]. The recommended MRI protocol includes conventional T1/T2, FLAIR, DWI, and a Gd-enhanced T1 sequence [4]. If there is concern for CNS vasculitis or cerebrovascular disease, then one might also consider MRA or brain angiography.

There are no specific findings on imaging that are diagnostic for NPSLE, but certain lesions, such as white matter hyperintensities, are seen in certain NPSLE syndromes. There have been studies examining WMH characteristics, looking for specific findings to relate to NPSLE. One study determined that NPSLE patients showed a higher periventricular or confluent WMH volume and more complex shape than non-SLE patients, being more significant in inflammatory NPSLE [25]. Another study looked at quantifying WML characteristics and showed that adding these details could enhance NPSLE diagnostics [26].

Advanced neuroimaging may need to be considered if conventional MRI does not reveal a cause of symptoms, and it should only be considered based on availability and local expertise [4]. These advanced techniques may reveal additional white or gray matter abnormalities, which still may not be specific to NPSLE but may help rule out other causes.

Advanced imaging, including quantitative MRI, magnetization transfer imaging, diffusion tensor MRI, perfusion-weighted imaging, radionuclide brain scanning (single photon emission computed tomography (SPECT)), or positron emission tomography (PET) can be considered [4,27]. Since no single technique covers all brain pathology, considering a multimodal approach combining structural imaging with functional or quantitative testing (MRI and SPECT) may be beneficial [28].

#### 3.3.3. Other Testing

Additional testing should be considered based on the specific manifestation of concern for NPSLE. EEG should be considered in patients with recurrent seizures. EMG or NCV should be considered in patients with peripheral neuropathy. Neuropsychological testing such as the Montreal Cognitive Assessment Questionnaire (MoCA) is useful to assess cognitive impairment in SLE patients, and the CES-D questionnaire and HADS anxiety questionnaire are useful for screening anxiety and depression [29]. Brain biopsy is not recommended for NPSLE but may be useful to exclude other conditions such as CNS lymphoma. Table 1 highlights a useful workup to consider based on NP manifestation.

### 3.4. Attribution Models

Given the challenges of diagnosing NPSLE, attribution models have been created to help clinicians if there is uncertainty over whether NP manifestation is due to SLE. The Italian Society of Rheumatology developed an attribution model based on a numerical algorithm from 0 to 10 [30] that has been validated [30,31].

This model addresses four themes:

1. The temporal relationship of NP events to the diagnosis of SLE (score: 0–3);
2. Identification of minor or common NP events [19] (score 0–3);
3. Recognition of confounding factors according to ACR case definitions (score > 1 factor = 0; 0 factors = 2);
4. Favoring factors (derived from EULAR recommendations for NPSLE) (score of 0 = none, up to 2 if >1 factor).

Favoring factors include major NP manifestation, generalized disease activity, previous major NPSLE events, +APLAs, MRI with WMH or more specific findings, CSF with lymphocytic pleocytosis, increased protein, and negative cultures [4]. A score greater than seven showed the best combination of sensitivity and specificity (87.9% and 82.6%, respectively) for attributing NP symptoms to NPSLE and had good performance when compared with expert judgment [24,30,31].

## 4. Pathogenesis

The overall pathogenesis of NPSLE cannot be explained by a single process, and there are various factors involved, including inflammatory injury or vascular injury among various genetic, environmental, and neuroendocrine factors [5,13,29,32].

The two main proposed pathways are as follows:

1. Autoimmune/inflammatory pathway: Pro-inflammatory mediators or autoantibodies against neuronal cells cause damage due to intrathecal immune complex formation and disruption of the blood-brain barrier. Manifestations with optic neuritis, transverse myelitis, peripheral neuropathy (mono multiplex), recurrent seizures, and diffuse manifestations may be caused by this pathway.
2. Vascular/ischemic/thrombotic pathway: Autoantibodies mediate vascular injury, causing cerebral microangiopathy, vascular occlusion, and hemorrhaging. Complement activation and deposition, accelerated atherosclerosis, coagulopathy, and immune complex deposition all contribute to damage in this pathway as well. Manifestations with positive APLAs, including cerebrovascular disease, chorea, seizures, myelopathy, and cognitive dysfunction, may be caused by this pathway.

Both pathways may occur at the same time and can cause focal and diffuse CNS along with PNS manifestations [32,33].

Several proinflammatory cytokines, including B-cell activating factor (BAFF), TNF-like weak inducer of apoptosis (TWEAK), IFN alpha, IFN gamma, and IL-2, 6, 8, and 10, have been implicated in pathogenesis, but more investigations need to be carried out to define their exact role [5,32,34].

Many autoantibodies have been studied, but none have been shown to be directly pathologic in NPSLE. APLAs have been recognized as a risk factor of NPSLE and cause direct antibody-mediated thrombosis, which leads to many manifestations of NPSLE, including stroke, seizures, and cognitive dysfunction [34]. Anti-ribosomal P antibodies occur in up to 46% of SLE patients but have not been shown to have a pathogenetic role in humans. Experimental data in mice and longitudinal association studies show a possible association with diffuse NPSLE, like psychosis [5,35,36], but clinical significance remains controversial [13]. Anti-NMDA antibodies (anti-N-methyl-D-aspartate receptor antibodies) occur in 30–40% of SLE patients and have been shown to be a subset of anti-dsDNA antibodies which cross-react with NMDA receptor subunit 2 (NR2), leading to neuronal death. There have been correlations between CSF anti-NMDA antibodies and diffuse NPSLE [34,36]. Anti-ribosomal and anti-NR2 antibodies induce neuronal cell death when passing through a disrupted BBB and are thought to be associated with diffuse NPSLE presentations [32,34]. Endothelial cell activation can lead to increased leukocyte adhesion, coagulation activation, and vascular thrombosis. Anti-endothelial cell antibodies (AECAbs) may contribute to BBB dysfunction by causing endothelial injury [13].

Many novel antibodies have been studied to look for an association with NPSLE, including anti-suprabasin, anti-UCH-L1, anti-BCRNA, and anti-GAPDH [32].

Blood-brain barrier (BBB) permeability increases in NPSLE, as indicated by CSF analysis of NPSLE patients showing increased immunoglobulins and pro-inflammatory cytokines, which may allow proteins to enter from blood circulation to cause intrathecal damage, but the exact mechanism is not well understood. Of note, local intrathecal antibody production may take place as well.

The complement system seems to have a role in disruption of the BBB, specifically C5a/C5aR. A study by Cohen et al. [37] compared brain histopathology in patients with NPSLE, SLE, and controls and demonstrated that NPSLE lesions ranged from focal vasculopathy to more specific lesions, including C4d- and C5b-9-associated microthrombi and diffuse vasculopathy.

Genetic factors including mutations in TREX1, a gene that encodes DNase III, have been seen in NPSLE patients among other diseases such as Aicardi-Goutieres syndrome, familial chilblain lupus, retinal vasculopathy, and cerebral leukodystrophy. Polymorphisms in this gene are associated with CNS manifestations, like seizures. Also, HLA-DRB1*04 may play a role, as it has been shown to correlate with disease activity in SLE patients [13,34,36].

While the pathogenesis is not well defined, focusing on the two proposed pathways can help understand clinical manifestations and potential effective treatments.

## 5. Treatment

Treatment of NPSLE depends on the underlying cause of the active NP manifestation and focuses on immunosuppressive treatment for autoimmune and inflammatory manifestations and anti-thrombotic therapy for vascular manifestations, along with symptomatic therapies. This can be challenging if it is not clear if the presenting manifestation is inflammatory or vascular in nature, and sometimes both can be present. There is a lack of high-quality studies performed on the treatment of NPSLE, but the EULAR created NPSLE management recommendations in 2010, using an evidence-based approach and expert consensus to help guide physicians [4].

### 5.1. General Lupus Management

All lupus patients should receive smoking cessation counseling and contraceptive counseling if applicable. SLE patients are at a higher risk of cardiovascular disease, and this should be discussed with the patients too. All patients should also be given antimalarial treatment, such as hydroxychloroquine, as a part of their lupus treatment, as long as there are no contraindications. Hydroxychloroquine has many benefits for SLE, including reduced accrual and damage, decreasing cardiovascular risk, and antithrombotic effects [38].

When treating SLE, all affected organ systems are taken into account, and an immunosuppressive regimen is created that will cover all active manifestations, where more serious, internal organ, or life-threatening manifestations would take precedent.

### 5.2. NPSLE Treatment

#### 5.2.1. Immunosuppressive Treatment

After secondary causes are excluded, immunosuppressive treatment is indicated if NP manifestations are found to reflect an inflammatory process, such as an acute confusional state, aseptic meningitis, myelitis, cranial and peripheral neuropathies, and psychosis [4]. It is important to note that some manifestations such as mild cognitive impairment and headaches may not need any immunosuppression.

Factors favoring the use of immunosuppressive treatment include NPSLE occurring close to SLE diagnosis, increased generalized lupus disease activity or flares, high scores in the NPSLE attribution algorithm, cerebrovascular events with negative APLAs (after exclusion of other embolic causes), evolving NP manifestations not responding to symptomatic treatments, relapsing NPSLE, moderate-to-severe neuro deficits, inflammatory CSF, or improvement with steroid trials [24].

First-line therapy is glucocorticoids, with the dosage depending on how severe the manifestation is. Prednisone, methylprednisolone, and dexamethasone are all synthetic glucocorticoids that have been used for NPSLE [13]. For severe, life-threatening manifestations, pulse dose glucocorticoids are administered, with 1000 mg of IV methylprednisolone (IV MP) daily for 3–5 days followed by a glucocorticoid taper, depending on the rate of improvement. For manifestations that are mild or moderate, IV or oral glucocorticoids may suffice at lower doses.

Very few randomized control trials have been performed for immunosuppressive treatment of NPSLE with only IV cyclophosphamide. A study by Barile-Fabris et al. [39] showed an induction regimen of 3 g of IV MP followed by monthly IV cyclophosphamide (IV CYC; 0.75 g/m^2^) versus IV MP bimonthly every 4 months for 1 year and then IV CYC or IV MP every 3 months for another year. The IV CYC regimen was seen to be superior, with clinical response in 18/19 patients compared with 7/13 patients who received IV MP alone. The patients in this study had NP manifestations of recurrent seizures, optic neuritis, peripheral or cranial neuropathy, coma, brainstem disease, or transverse myelitis. IV CYC should be considered for induction therapy in severe manifestations of NPSLE.

Many other immunosuppressive medications have been used for treatment, with only low-quality evidence available in the literature for NPSLE. Mycophenolate mofetil (2–3 g/d) has also been used for NPSLE syndromes as well [24,40]. Other immunosuppressive agents used include azathioprine (2 mg/kg/d), which is often utilized for maintenance therapy in mild-to-moderate manifestations of NPSLE [40]. Other treatments used for immunosuppressive manifestations include methotrexate, cyclosporine, rituximab, plasma exchange, and IVIg [24,38,40]. A study reviewing short- and long-term follow-ups after immunosuppressive treatment, which was usually glucocorticoids with cyclophosphamide induction therapy followed by azathioprine maintenance therapy, showed improvement in symptoms in 70% of events [41]. A study by Bortoluzzi et al. [42] retrospectively reviewed 350 NP events to investigate therapeutic strategies and disease outcomes for patients experiencing their first NP manifestations and found that glucocorticoids and immunosuppression were more frequently used for central diffuse or focal NP manifestations, with 64% showing clinical improvement at a 12 month follow-up.

Newer therapies approved for SLE include belimumab and anifrolumab. Belimumab is an anti-BAFF medication recently approved for lupus nephritis in conjunction with standard therapy in 2020 [43]. Anifrolumab, a type-1 IFN gamma receptor antagonist, was FDA-approved for the treatment of SLE in 2021 [44]. Unfortunately, patients with NPSLE were not included or formally evaluated in trials, and thus its use in NPSLE is unknown. Side effects of belimumab include depression, suicide, and self-harm. One study showed no clear protection from belimumab treatment used for SLE patients without active NPSLE [21].

#### 5.2.2. Ischemic Treatment

Ischemic manifestation treatment includes antiplatelet or anticoagulation therapy.

Factors favoring the use of anti-thrombotic treatment include positive moderate-to-high titers of APLAs, cerebrovascular disease with +APLAs, MRI with ischemic or thrombotic lesions in the context of atherosclerotic risk factors or +APLAs, high cardiovascular risk, and NP manifestations of a presumed ischemic cause not responding to immunosuppression [24]. Antiplatelet agents may be considered for primary prevention in SLE patients with persistently positive moderate or high APLA titers who have not experienced an NP event.

In patients who have had an ischemic stroke and fulfill the classification criteria for APS, they should receive long-term anticoagulation for secondary prevention of recurrent strokes. In APS, warfarin is the preferred long-term anticoagulant, with a target INR of 2–3 for secondary thrombosis prevention. More data are needed for direct oral anticoagulants in the treatment of APS. Anticoagulation should also be considered in manifestations strongly associated with APLAs, like seizures and chorea. Of note, while anticoagulation is a critical part of APS management, patients also often require immunosuppression [4,11]. Patients with ischemic manifestations with negative APLAs likely do not need anticoagulation and can be managed with anti-platelet therapy and lipid lowering therapy [24].

#### 5.2.3. Symptomatic Treatment

Depending on the manifestations present, symptomatic therapy may be warranted, including antiepileptics, antipsychotics, anxiolytics, and mood stabilizers [4,13]. One study compared memantine use in SLE patients with cognitive impairment and found no significant improvement when compared with a placebo group, except for controlled oral word association [45].

#### 5.2.4. Therapeutic Challenges

**Figure 1:**
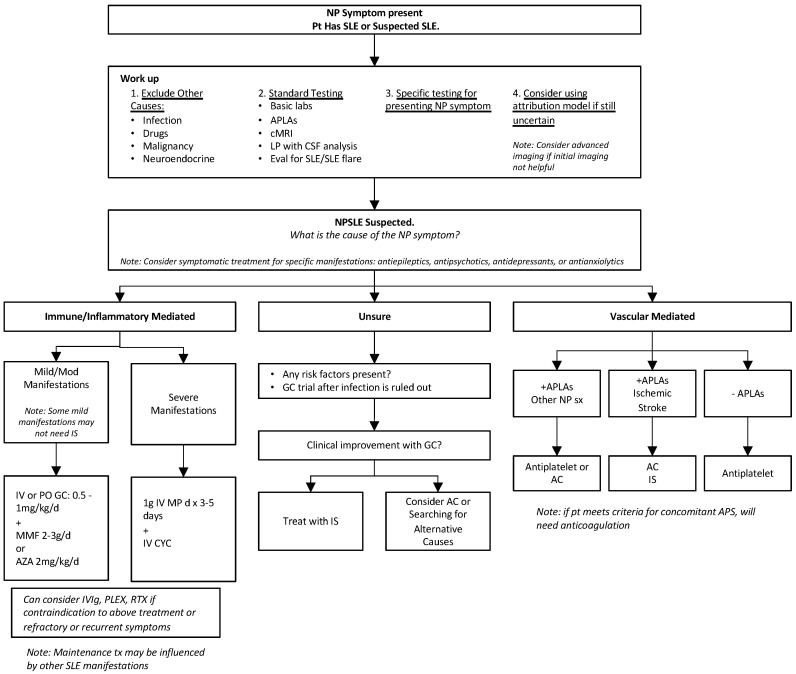
Diagnostic and therapeutic approach to suspected NPSLE. NP = neuropsychiatric; APLAs = antiphospholipid antibodies; cMRI = conventional magnetic resonance imaging; LP = lumbar puncture; CSF = cerebrospinal fluid; GC = glucocorticoid; MMF = mycophenolate mofetil; AZA = azathioprine; MP = methylprednisolone; CYC = cyclophosphamide; IS = immunosuppression; AC = anticoagulation; IV = intravenous; PO = per os.

While current treatment strategies rely on the ability to differentiate between inflammatory and vascular etiology of manifestations, in clinical practice, it may be unclear which etiology is at play, especially for cerebrovascular events, which can be caused by inflammatory and ischemic mechanisms. In cases of uncertainty, a glucocorticoid trial is a reasonable approach once infection is ruled out, and if there is a clinical response, then further immunosuppression can be considered. Figure 1 shows a diagnostic and treatment algorithm that may be helpful when suspecting NPSLE.

## 6. Juvenile-Onset NPSLE

Children with SLE are also at risk of developing neuropsychiatric symptoms, termed childhood or juvenile-onset NPSLE (jNPSLE), when diagnosed before the age of 18. The ACR case definitions for NPSLE syndromes are applied to pediatric patients when a diagnosis of jNPSLE is being considered [2]. The majority of patients are female, and the median age at NP involvement is 14 years old [46]. Similar to adult-onset NPSLE, jNPSLE may occur at any time in the disease’s course but most commonly within the initial year of presentation, and it can include central or peripheral neurologic syndromes. The most common neurologic presentations are headaches, cognitive dysfunction, seizures, cerebrovascular disease, and movement disorders. The most common psychiatric disorders include mood disorders, psychosis, and anxiety [47]. Many patients have more than one NP manifestation at presentation. Juvenile NPSLE patients have additional SLE symptoms, such as renal involvement, chilblains [48], or hematologic, skin, and serositis manifestations [46]. Treatment strategies are similar to those for adult NPSLE management.

## 7. Future Directions

The many challenges faced today regarding NPSLE are opportunities for future directions and advancements. Firstly, more high-quality studies are needed with current available therapeutics to help guide treatment with more evidence. More specific biomarkers and imaging modalities are needed to help guide diagnosis. Many biomarkers are being studied currently, including TNF-like weak inducer of apoptosis (TWEAK), lipocalin 2 (LCN2, an iron transporter in innate immunity), S100B (strongly associated with peripheral neuropathy), and brain-derived neurotrophic factor (BDNF). Hopefully, investigations into these biomarkers will lead to better insights into pathogenesis or potential therapeutic targets. Other opportunities for therapeutic targets include pro-inflammatory cytokines such as IL-6 or type-1 IFN gamma. Complement depositions, specifically C5aR, are seen in ischemic pathogenesis and can also be a potential drug therapy target.

Angiotensin-converting enzyme (ACE) has been shown to regulate brain function and inflammation, and studies using ACE inhibitors in mice have shown decreased type I interferon response and improved cognitive deficits, showing possible potential in the neuroprotective effects of ACE inhibitors [24,29,33].

## 8. Conclusions

NPSLE presents a multifaceted challenge in clinical rheumatology, encompassing a spectrum of neurologic and psychiatric symptoms that significantly impact a patient’s quality of life. This review underscores the complexities of NPSLE diagnosis and management, emphasizing the need for a multidisciplinary approach incorporating advances in diagnostic criteria, imaging techniques, and a clearer understanding of underlying pathogenic mechanisms. Moving forward, it is imperative that continued research focuses on evaluating diagnostic biomarkers and novel targeted therapies. In the future, hopefully, this will lead to better diagnostic accuracy and effective management.

## Figures and Tables

**Table 1:** NPSLE syndromes as defined by ACR [2] and diagnostics to consider for specific manifestations.

Focal		Diffuse	
**Central NS**	Helpful Diagnostics to Consider	**Central NS**	Helpful Diagnostics to Consider
Aseptic Meningitis	LP and MRI	Acute Confusional state	LP and MRI to exclude infection Neuropsych testing
Cerebrovascular Disease	EKG, echocardiogram, carotid doppler MRA and LP (if concern for CNS vasculitis)	Anxiety	
Demyelinating Syndromes	LP and MRI Consider testing for MS	Cognitive dysfunction	Neuropsych testing
Headaches		Mood disorder	Psychiatric evaluation
Movement Disorders: Chorea		Psychosis	Psychiatric evaluation
Myelopathy	Gad-enhanced MRI and LP and CSF analysis		
Seizure Disorder	MRI, EEG, LP Important to exclude structural brain disease and inflammatory or metabolic conditions, infections		
**Peripheral NS**	Helpful Diagnostics to Consider		
AIDP	EMG and NCV, LP		
Autonomic Disorder			
Mononeuropathy (Single or Multiplex)	EMG and NCV		
Myasthenia Gravis	CT exclude thyroid disease, specific ab testing (AchR, MuSK, LRP4)		
Plexopathy			
Polyneuropathy	EMG and NCV		
Cranial Neuropathies			

LP: lumbar puncture, MRI: magnetic resonance imaging, EKG: electrocardiogram, MRA: magnetic resonance angiography, MS: multiple sclerosis, CSF: cerebrospinal fluid, EEG: electroencephalogram, EMG: electromyography, NCV: nerve conduction velocity, CT: computed tomography, AchR: acetylcholine receptor antibody, MuSK: muscle specific kinase antibody, LRP4: low density lipoprotein receptor-related protein 4 antibody.
